# Irreversible intraocular pressure elevation as a complication of MIRAgel scleral buckling

**DOI:** 10.1016/j.ajoc.2022.101583

**Published:** 2022-05-13

**Authors:** Yuya Tankyo, Yosuke Harada, Tomona Hiyama, Hiromi Ohara, Mina Mizukami, Hideaki Okumichi, Kazuyuki Hirooka, Yoshiaki Kiuchi

**Affiliations:** Department of Ophthalmology and Visual Science, Graduate School of Biomedical Sciences, Hiroshima University, 1-2-3 Kasumi, Minami-ku, Hiroshima City, Hiroshima, 734-8551, Japan

**Keywords:** MIRAgel, Ocular hypertension, Scleral buckle, Glaucoma, Hydrogel explant, Retinal detachment

## Abstract

**Purpose:**

To report a case of ocular hypertension due to swelling and degeneration of hydrogel explant (MIRAgel) after retinal detachment surgery.

**Observations:**

The patient who had a history of left retinal detachment 23 years prior had been regularly followed up for epiretinal membrane in the left eye at the Department of Ophthalmology, Hiroshima University Hospital. Two years after the first presentation, the patient had symptoms of foreign body sensation and hyperemia, with elevation of the intraocular pressure (IOP) of the left eye to 24 mmHg. Two months later, the patient noticed omnidirectional oculomotor disturbances in the left eye, and magnetic resonance imaging (MRI) revealed swelling of the buckle material, presumably hydrogel explant, surrounding his left eye. His oculomotor disturbances worsened, and the left eye IOP remained high at 40 mmHg, despite the administration of antihypertensive eye drops. Subsequently, the swollen hydrogel explant was surgically removed. After the surgery, there was improvement of the diplopia and foreign body sensation. However, IOP in the left eye remained at 34 mmHg, and a trabeculectomy was performed to normalize the IOP.

**Conclusions and Importance:**

As far as we know, there have been no reported cases of irreversible ocular hypertension due to hydrogel explant. Stenosis of the trabecular outflow pathway secondary to compression of the superior scleral vein by long-term swollen hydrogel explant and inflammation around the hydrogel explant may be the cause of irreversible IOP elevation. Trabeculectomy may be effective for treating the intraocular hypertension caused by hydrogel explant.

## 1 Introduction

Hydrogel explant (MIRAgel®︎, MIRA Inc., Waltham, MA) was introduced as a new scleral explant for treating rhegmatogenous retinal detachment and was widely used in retinal restoration surgery from 1985 to early 1997.^1^ Initially, it was thought to be an ideal material because of its pliable characteristics and ability to absorb and release antibiotics, thereby minimizing scleral erosion or postsurgical infection.^2^ However, severe complications caused by buckle hydrolysis and expansion were revealed during the long-term follow-ups, which resulted in discontinuation of this buckle material.^3^^,^^4^

The complications of hydrogel explant were associated with swelling of the material, which leads to limited extraocular motility, rectus palsy, strabismus, ptosis, hyperemia, protrusion of the buckle beneath the eyelid, infection around the buckle and scleral perforation.^1^^,^^5^ However, as far as we know, there have been no reports regarding changes in the IOP secondary to the buckle swelling. Here, we report a case of irreversible intraocular hypertension that was presumably caused by hydrogel explant swelling that subsequently required trabeculectomy.

## 2 Case report

**Fig. 1:**
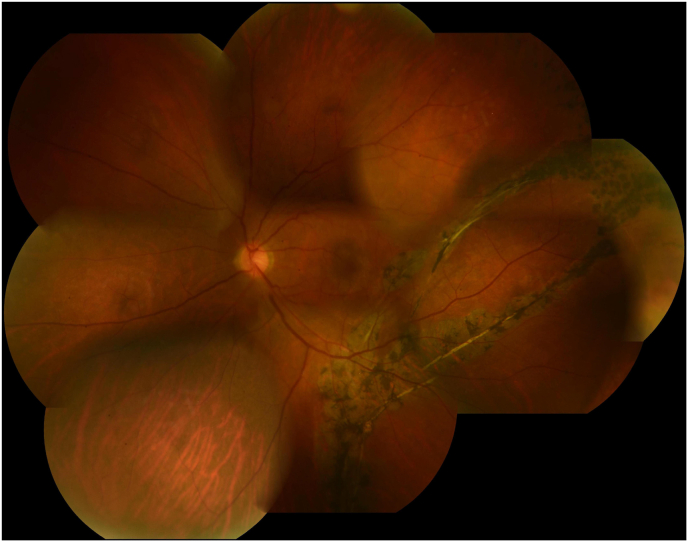
Fundus photograph in the left eye at the first visit. There is broad retinal atrophy with subretinal strands due to old retinal detachment which was treated with scleral buckling.

A 56-year-old male patient presented with decreased visual acuity in his left eye. He had a history of left retinal detachment 23 years prior, which was treated at other hospital. At presentation, his decimal best corrective visual acuity (BCVA) was 1.0 in the right eye and 0.9 in the left eye. IOPs of the right and the left eyes were 12 mmHg and 11 mmHg, respectively. Although ocular motility showed orthophoria in the primary position with a slight adduction limitation in the left eye, he did not have double vision. Slit-lamp examination showed no inflammation in either of his eyes. Fundus examination of the right eye revealed lattice degeneration surrounded by laser retinopexy in the superotemporal retina. The left eye exhibited epiretinal membrane at the macular and scleral indentation secondary to the buckling element in the inferotemporal retina with broad chorioretinal atrophy (Fig. 1). As epiretinal membrane was assumed to be the possible cause of the decreased visual acuity in the left eye, the patient was regularly monitored by retina specialists.

**Fig. 2:**
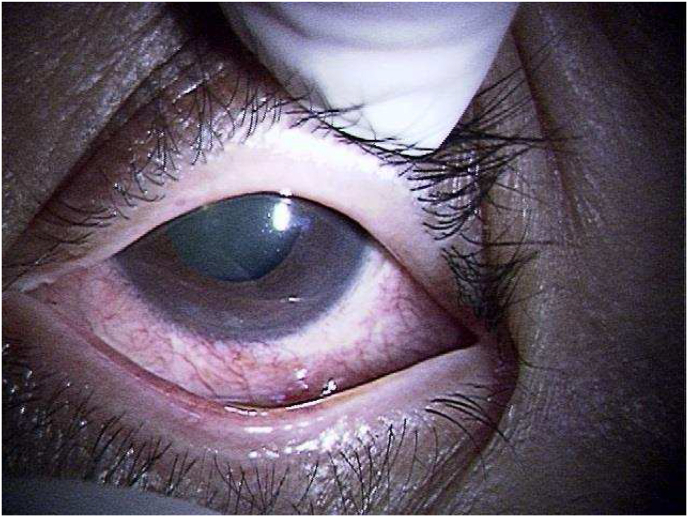
Slit-lamp photographs of the left eye at two years after the first visit. There was predominant hyperemia inferiorly, and the cornea and anterior chamber were clear.

**Fig. 3:**
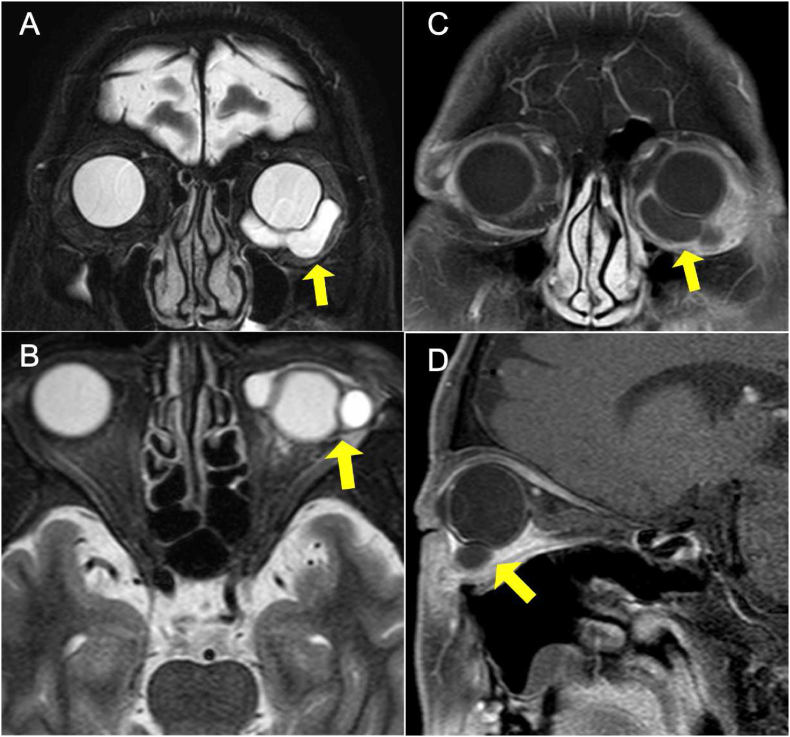
Contrast-enhanced T2-weighted MRI images of the orbit in (A; coronal) and (B; axial), and contrast-enhanced T1-weighted images (C; coronal) and (D; sagittal). There was no external ocular muscle hypertrophy or mass lesions in the bilateral orbits. Well-circumscribed buckle material found to have T1 hypointensity and T2 hyperintensity that was consistent with a fluid signal was observed around the left eye. The high intensity signal observed around the buckle material and subcutaneous in the left eyelid by T1-weighted fat-saturated post gadolinium suggested inflammation.

**Fig. 4:**
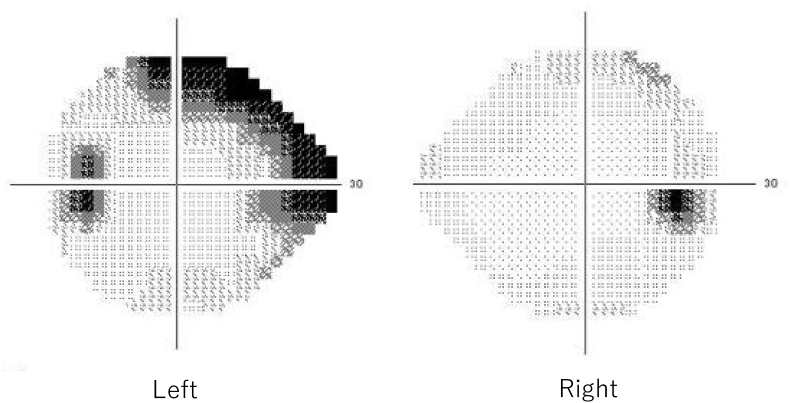
The 24-2 Hamphrey visual field test result revealed normal in the right eye and superonasal defect in the left eye.

**Fig. 5:**
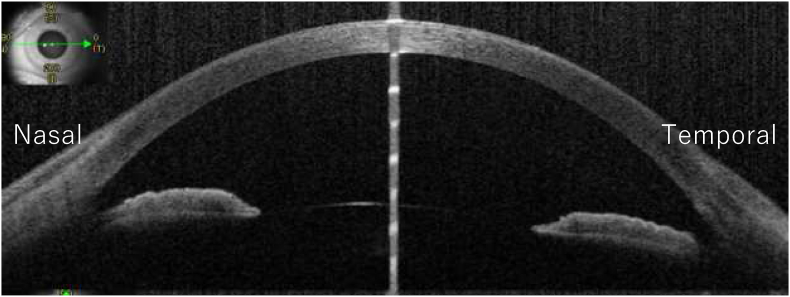
Anterior segment optical coherence tomography (coronal section across the green arrow from nasal to temporal) in the left eye before removing the hydrogel explant. Anterior chamber angle image was open before the removal of the hydrogel explant. (For interpretation of the references to colour in this figure legend, the reader is referred to the Web version of this article.)

**Fig. 6:**
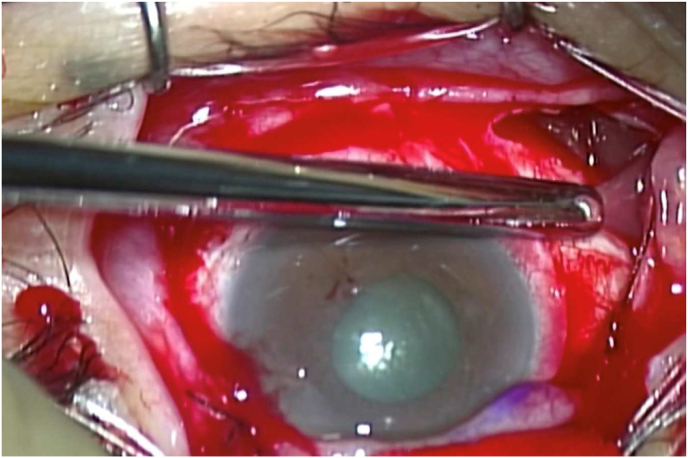
Intraoperative view of hydrogel explant removal surgery. The swollen hydrogel explant was difficult to grasp and remove with forceps, as it had become brittle. Thus, it was removed using a suction cannula.

Two years after the first presentation, he noticed hyperemia and complained about foreign body sensations in his left eye. BCVA was 1.0 in the right eye and 0.7 in the left eye. IOPs in the right and the left eye was 12 mmHg and 24 mmHg, respectively. A limitation of adduction and supra-adduction of the left eye was observed. Slit-lamp examination showed that he had hyperemia with a clear cornea and quiet anterior chamber in the left eye (Fig. 2). Fundus examination of the left eye did not remarkably change from that observed during the first visit. To assess the restriction of extraocular movements and hyperemia in the left eye, magnetic resonance imaging (MRI) was performed. On MRI, well-circumscribed buckle material that demonstrated T1 and T2 hyperintensity and which was consistent with a fluid signal was found around the left eye (Fig. 3). In addition, a high-intensity signal around the buckle material and subcutaneous area in the left eyelid was observed by T1-weighted fat-saturated post gadolinium. Based on these MRI findings, it was assumed that the buckle material was likely hydrogel explant, and that his symptoms, such as restricted extraocular movement and hyperemia, were caused by the swollen hydrogel explant and inflammation that occurred around this area. Since the patient did not want to undergo buckle removal at this point, he was closely monitored in conjunction with the use of steroid (0.1% fluorometholone eye drop) and anti-hypertensive eye drops. However, there was gradual elevation of his left IOP to 40 mmHg despite the use of hypotensive agents (latanoprost, dorzolamide hydrochloride, and timolol maleate) and superonasal defect in the left eye was revealed by the 24-2 Hamphrey visual field test (Fig. 4). Slit-lamp examination of the left eye demonstrated the presence of hyperemic perilimbal vessels (ciliary flush), although there was no evidence of inflammation in the anterior chamber. Furthermore, gonioscopy and anterior segment optical coherence tomography revealed an open angle without peripheral anterior synechiae (Fig. 5). The IOP remained high as 38 mmHg even after the discontinuation of steroid eye drops. The IOP in his left eye was thought to be related to the swollen hydrogel explant, as the IOP elevation developed in conjunction with his other ocular symptoms that were associated with the hydrogel explant. Subsequently, the hydrogel explant was surgically removed under general anesthesia as previously described.^6^ Briefly, the conjunctiva and capsule around the implant were incised by ophthalmic scissors, with a suction cannula used to aspirate the degraded hydrogel implant (Fig. 6). After removal of the hydrogel explant, the restriction of the extraocular movement improved. However, the high IOP persisted, with the pressure remaining around 40 mmHg, even in conjunction with the use of antihypertensive drugs (latanoprost, dorzolamide hydrochloride, timolol maleate). Seven days after hydrogel explant removal, trabeculectomy of the left eye was performed. After the surgery, there was a choroidal detachment in the left eye due to hypotony (2 mmHg), which resolved 2 weeks after the surgery with 0.1% dexamethasone eye drops. The IOP in the left eye then stabilized between 5 and 8 mmHg without anti-glaucoma eye drop and steroid eye drop.

## 3 Discussion

Previous reports have discussed the various complications that can occur after hydrogel explantation.^1^^,^^5^ While these common complications are normally limited to extraocular motility, rectus palsy, strabismus, ptosis, hyperemia, protrusion of the buckle beneath the eyelid, and scleral perforation due to buckle swelling, the irreversible IOP elevation caused by hydrogel explant is not well known. In this case report, we present a patient with IOP elevation that occurred 23 years after hydrogel explantation, which did not improve after the buckle removal, thereby requiring a trabeculectomy to normalize the IOP.

The most common buckle-related IOP elevation is due to angle closure glaucoma without pupil block.^7^ The anterior chamber becomes shallow due to forward displacement of the ciliary body, which is thought to be caused by the combined effects of an interrupted choroidal venous drainage along with the mass effect of a large explant. This usually occurs during the early postoperative period, with resolution occurring after one week in conjunction with conservative treatment that includes steroid, cycloplegia, and ocular hypotensive agents. In contrast, Pinninti et al. evaluated patients who were followed for at least ten years after undergoing the encircling scleral buckling procedure and found that there was no evidence of long-term risk for developing glaucoma in eyes with encircling scleral buckling.^8^ In our case, the IOP elevation occurred more than 20 years after the initial buckling surgery, and gonioscopy revealed an open angle without peripheral anterior synechiae in the eye. Nevertheless, this was assumed to be associated with the hydrogel implant rather than the primary open angle glaucoma, as it was accompanied by deterioration of eye movement, hyperemia, and foreign body sensation. The plausible explanation for the IOP elevation in this case was obstruction of the trabecular outflow pathway caused by the swollen hydrogel explant. Elevated episcleral venous pressure has been shown to be associated with an elevated IOP.^9^ Thus, in our current case, the swelling and degeneration of the hydrogel explant might have compressed the sclera over a long time, thereby resulting in stenosis of the superior scleral vein, and subsequently leading to the obstruction of the uveoscleral outflow and elevation of the episcleral venous pressure. These changes might have then prevented the drainage of the aqueous humor and caused an increased IOP. Another possibility for the IOP elevation could have been scleral inflammation.^10^ It has been reported that inflammation in the sclera can cause IOP elevation.^10^^,^^11^ The sclera around the hydrogel explant was probably under the prolonged inflammation due to the swollen hydrogel explant, causing ciliary flush the left eye. This suggests that the inflammation might have been related to the IOP elevation. It remains unclear as to why there have been no reports on IOP elevations as a complication of hydrogel explant. One possibility is that the hydrogel expansion may more strongly affect eye movement as compared to the scleral compression, enabling patients to be aware of their ocular movement or ptosis as a complication prior to the development of the IOP elevation.

Surgical treatment for glaucoma after scleral buckling is challenging because the conjunctiva is scarred by the original buckle procedure. Thus, the 2002 Survey of the American Glaucoma Society reported that a glaucoma drainage device was preferred to performing trabeculectomy in patients with previous scleral buckling.^12^ However, trabeculectomy was chosen in our current patient for the following reasons. First, there was concern regarding the deterioration in the limitation of the eye movement and the diplopia that occurred in conjunction with the glaucoma drainage device. Second, there was no evaluation of the condition of the sclera that had been indented for a long period of time with regard to whether a glaucoma drainage device could be safely attached to the sclera. Third, the superotemporal conjunctiva appeared to be intact and mobile enough to perform trabeculectomy. However, in these particular cases, the superior scleral venous pressure may be elevated. Thus, it is advisable to use an anterior chamber maintainer, as choroidal detachment and choroidal hemorrhage are known complications of trabeculectomy. Based on these issues, we decided to perform a trabeculectomy, which was conducted without any complications. As a result, the IOP in the left eye was normalized without causing extraocular dysmotility or diplopia.

## 4 Conclusions

Irreversible IOP elevation is a rare complication of hydrogel explant. Stenosis of the trabecular outflow pathway secondary to compression of the superior scleral vein by long-term swollen hydrogel explant and inflammation around the hydrogel explant may be the cause of irreversible IOP elevation. Trabeculectomy may be effective for treating the intraocular hypertension caused by hydrogel explant.

## Patient consent

Informed consent was obtained from the patient for the purpose of publication.

## Funding

No funding or grant support.

## Authorship

All authors attest that they meet the current ICMJE criteria for Authorship.

## Declaration of competing interest

All authors have no financial disclosures.
